# Molecular Imaging of Aminopeptidase N in Cancer and Angiogenesis

**DOI:** 10.1155/2018/5315172

**Published:** 2018-06-25

**Authors:** Cynthia L. Schreiber, Bradley D. Smith

**Affiliations:** Department of Chemistry and Biochemistry, University of Notre Dame, 236 Nieuwland Science Hall, Notre Dame, IN 46556, USA

## Abstract

This review focuses on recent advances in the molecular imaging of aminopeptidase N (APN, also known as CD13), a zinc metalloenzyme that cleaves *N*-terminal neutral amino acids. It is overexpressed in multiple cancer types and also on the surface of vasculature undergoing angiogenesis, making it a promising target for molecular imaging and targeted therapy. Molecular imaging probes for APN are divided into two large subgroups: reactive and nonreactive. The structures of the reactive probes (substrates) contain a reporter group that is cleaved and released by the APN enzyme. The nonreactive probes are not cleaved by the enzyme and contain an antibody, peptide, or nonpeptide for targeting the enzyme exterior or active site. Multivalent homotopic probes utilize multiple copies of the same targeting unit, whereas multivalent heterotopic molecular probes are equipped with different targeting units for different receptors. Several recent preclinical cancer imaging studies have shown that multivalent APN probes exhibit enhanced tumor specificity and accumulation compared to monovalent analogues. The few studies that have evaluated APN-specific probes for imaging angiogenesis have focused on cardiac regeneration. These promising results suggest that APN imaging can be expanded to detect and monitor other diseases that are associated with angiogenesis.

## 1. Introduction

Aminopeptidase N (APN; EC 3.4.11.2, also known as CD13) is a Zn^2+^-dependent membrane-bound enzyme that cleaves *N*-terminal neutral amino acids and is a useful target for molecular imaging [1]. APN was first purified in 1963 and later shown to be overexpressed in cancer [2], tumor angiogenesis [3], and cardiac angiogenesis [4]. Originally, APN was called aminopeptidase M referring to its location on the cell membrane. In 1980, the name was changed to APN, to highlight its specificity for *N*-terminal neutral amino acids [5]. Within the immunology community, APN is often referred to as the myeloid antigen CD13. It was discovered in 1989 that APN and CD13 were identical proteins, and now, the two names are used interchangeably [6]. In the last five years, there has been an increasing number of publications describing preclinical and clinical research on APN for imaging and therapeutics [7]. The purpose of this review was to summarize the recent advances in molecular imaging of APN for detection of cancer and angiogenesis.

APN is expressed in a range of different human cells such as macrophages, stromal cells, smooth muscle cells, and fibroblasts [8]. The enzyme has also been referred to as a moonlighting enzyme due to its involvement in peptide cleavage, viral infection, endocytosis, and cell signaling [9]. Unusually high levels of APN are found in various cancers including breast [8, 10], ovarian [11, 12], thyroid [13], pancreatic [14], colorectal [15], and NSCLC [16, 17]. In gastric cancer, APN and TGF-*β*1 expression levels were correlated with tumor size, lymph node metastasis, and tumor differentiation [18]. Similarly, in pancreatic cancer, the serum APN level correlated with tumor size, lymph node metastasis, and metastasis stage. It is a diagnostic and prognostic biomarker of early stage pancreatic cancer, predicting mortality and overall survival of pancreatic cancer patients [19]. Using tumor tissue and plasma from colorectal cancer patients, higher APN enzyme activity in tissue correlated with better overall survival. In contrast, higher APN enzyme activity in plasma led to worse overall survival. In these colorectal cancer patients, there was no correlation between APN enzyme activity in tumor tissue and plasma, but each factor could be used independently to predict patient's 5-year survival [20]. Another study evaluated the relationship between APN expression and osteosarcomas where immunohistochemistry showed that 77% of patients were APN positive. Furthermore, higher APN expression correlated with poor overall survival [21]. The APN expression in vasculature correlated with a poor overall survival in NSCLC patients diagnosed with tumor stage III and lymph nodes status pN2+. Thus, this subgroup of NSCLC patients was identified as candidates for APN-targeted imaging and therapy [22]. Taken together, the level of APN expression is a useful cancer biomarker for predicting clinical outcome. While overexpressed APN is a well-known cancer biomarker, it is worth noting that there are certain cancers such as renal cancers where the APN expression is decreased compared to the surrounding normal tissue [23]. In fact, it was shown that molecular imaging of kidney APN expression could provide pathophysiological information about kidneys noninvasively [24]. Despite the high expression in kidneys and some other normal tissues, APN is considered to be a promising candidate for cancer imaging and therapy if the probe structure is optimized to have ideal pharmacokinetic properties and targeted tissue uptake.

APN is not expressed on the surface of normal vasculature, but there is high APN expression on blood vessels undergoing angiogenesis such as tumor vasculature [3, 25]. This activation is controlled by angiogenic signals such as galectin-3 and Ras, where Ras is important in signal transduction cascades [26, 27]. When key signaling pathways were impaired, APN was sufficient to rescue angiogenesis. However, when APN was knocked out in normal mice, there was a severely impaired angiogenic response to pathological conditions. Interestingly, these mice were able to develop normally when no physiological alterations were applied [28]. Furthermore, when APN was knocked out in a mouse model of occlusive peripheral artery disease, there was impairment in healing and muscle regeneration despite a prohealing cytokine environment [29]. These studies reveal the important role APN plays in angiogenesis and suggest that molecular targeting of APN can be used to monitor cardiovascular diseases such as atherosclerosis, myocardial infarction, and peripheral artery disease.

## 2. Structure and Function of APN

**Figure 1:**
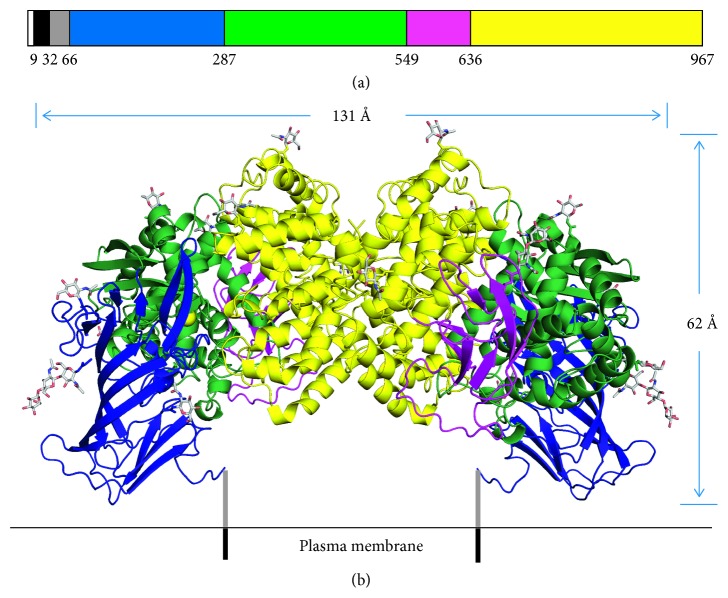
Structure of the APN enzyme. (a) The monomeric sequence is composed of 967 residues and 7 sections as designated by color. From left to right, the order is as follows: cytoplasmic domain, transmembrane section, Ser/Thr rich stalk, and ectodomain I, II, III, and IV. (b) The ribbon structure of how the dimeric APN enzyme is likely oriented on the plasma membrane. The yellow spheres are the active sites for the zinc ions, and the *N*-linked oligosaccharides are represented by sticks. Reprinted (adapted) with permission from Wong et al. [31], copyright (2012) from American Society for Biochemistry and Molecular Biology.

APN is part of the M1 family of zinc metalloenzymes [30]. At full length, human APN is composed of 967 residues and 7 sections, four of which are ectodomains (Figure 1) [31]. Amino acids 1–66 correspond to the cytoplasmic domain, transmembrane section, and the Ser/Thr rich stalk. Through the stalk, ectodomain I (66–287 aa) stabilizes the APN enzyme in the plasma membrane. Ectodomain II (287–549 aa) contains the zinc binding site and catalytic site, which is located in a large internal cavity inaccessible to the bulk solvent. Lastly, ectodomain IV (636-967 aa) is responsible for creating the dimer interface through hydrophobic interactions and a salt-bridge network.

**Figure 2:**
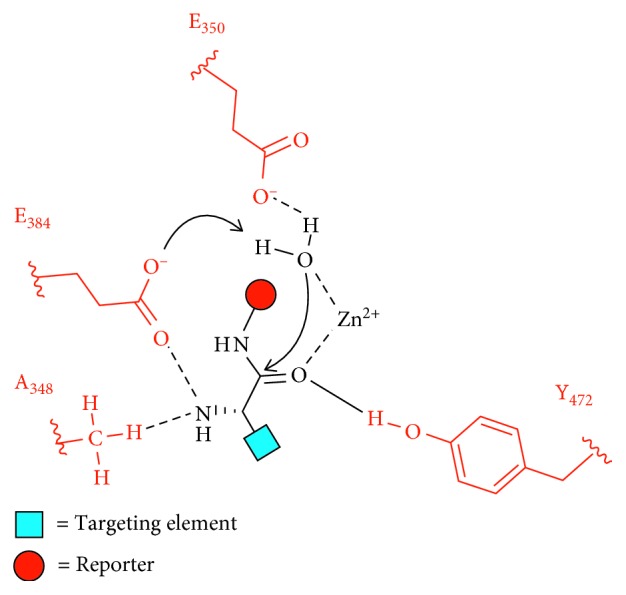
Endogenous cleavage of an enzyme substrate containing a targeting element and a reporter group in the enzymatic site of porcine APN. The substrate is oriented in the enzyme active site by residues A_348_, E_384_, and Y_472_. A catalytic water is activated by a zinc cation and E_350_. The water then attacks the substrate carbonyl, and E_384_ shuttles a proton from the catalytic water to the leaving nitrogen [33].

For enzyme activity, these 4 ectodomains coordinate together to create an open or closed conformation. The enzyme initially starts in an open conformation and allows the peptide substrate to travel through a negatively charged channel to reach the catalytic site. The *N* terminus of the peptide substrate then interacts with the catalytic site while the rest of the substrate interacts with the channel through hydrogen bonding. The APN enzyme then adopts a closed conformation to initiate peptide bond cleavage. This involves a 15° movement where ectodomains I through III swing over to ectodomain IV which allows for encapsulation/orientation of the substrate for catalysis [32]. Next, in porcine APN, a catalytic water is activated by a zinc cation, and the water is positioned by E350. The water then attacks the carbonyl carbon, and E384 shuttles a proton from the catalytic water to the leaving nitrogen (Figure 2) [33]. After catalysis, the APN enzyme converts to the open conformation for substrate release. In addition, each APN monomer can adopt either an open or closed conformation. It is unknown if the movement of each monomer is random or synchronized. However, when dimeric human APN was incubated with a substrate, ∼50% of the molecules were in different conformations, suggesting that each monomer has a distinct conformation [32].

## 3. Measuring APN Activity Using Reactive Substrates

**Figure 3:**
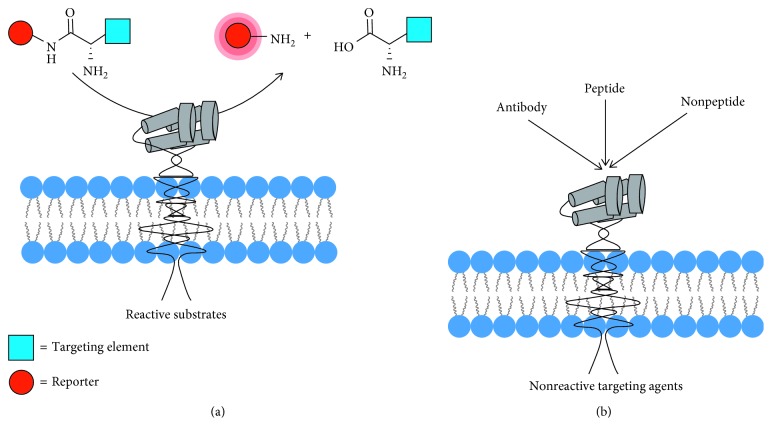
Types of APN-targeting agents. (a) The APN enzyme cleaves the *N*-terminal amino acid from reactive substrates to release an imaging reporter group. (b) Nonreactive targeting agents bind to the active site or the exterior surface of the enzyme and are not cleaved.

APN-targeting agents can be classified into two large subgroups: reactive substrates that are cleaved by the enzyme and nonreactive targeting agents (Figure 3). Reactive targeting agents (substrates) contain a reporter group that is released by the enzyme. Since APN has a preference for cleaving *N*-terminal neutral amino acids from peptides [9], most of the optically active substrates for APN are carboxyl-modified derivatives of L-leucine or L-alanine. The simplest colorimetric example is L-leucine-p-nitroanilide where the cleaved product, p-nitroaniline, absorbs at 405 nm. This commercially available molecule is often used for enzyme inhibitor assays but is limited by poor detection sensitivity [34, 35]. More sensitive fluorescent assays employ a L-leucine or L-alanine derivative that is cleaved enzymatically to release fluorescent 4-methyl-7-coumarinylamide (AMC). This assay has been employed to detect soluble APN in the intratumoral fluid of ovarian cancer patients and in the synovial fluid of rheumatoid arthritis patients [11, 36].

In recent years, there has been emphasis on substrates that release a deep-red or near-infrared (NIR) fluorescent dye that is better suited for clinical imaging and detection. One promising application is fluorescence-guided surgery where the surgeon aims to resect cancerous tissue with negative margins and improve patient treatment outcomes [37]. A recent study developed a NIR substrate, amino hemicyanine alanine, to image the enzymatic activity of APN in vitro and in vivo. Substrate cleavage by the APN enzyme produced an increase in NIR fluorescence and enabled imaging of APN-positive tumor-bearing mice models [38]. One way to further enhance detection sensitivity and attenuate artifacts due to environmental and instrument effects is to design a ratiometric enzyme substrate. A ratiometric APN substrate was recently produced by conjugating alanine to a cresyl violet fluorophore. Substrate cleavage induced a fluorescence change from 575 nm to 626 nm and permitted APN detection in cells and human urine samples with high sensitivity [39]. Another ratiometric enzyme substrate, alanine naphthalimide, identified APN enzymatic activity on ovarian cancer cells where there was a 90 nm red shift along with a distinct color change when the alanine was cleaved [40].

**Figure 4:**
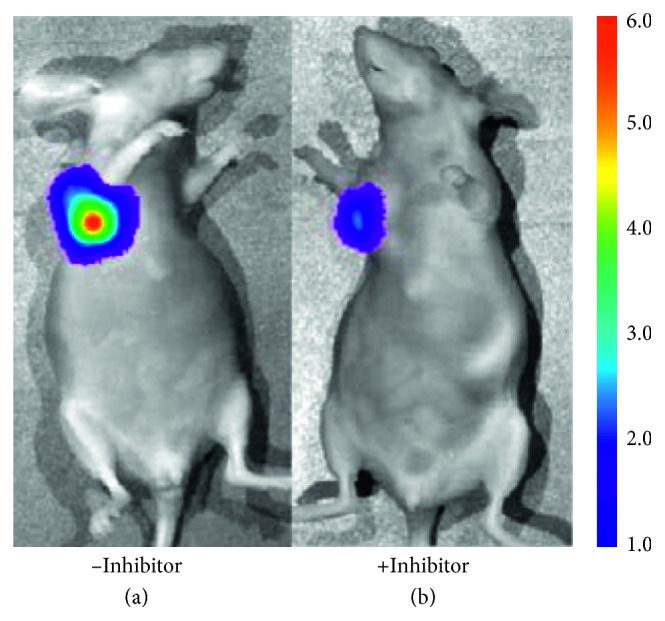
Bioluminescence imaging of APN enzyme activity in an ovarian cancer tumor mouse model. (a) Imaging with the L-leucine-aminoluciferin probe. (b) Blocked imaging of the L-leucine-aminoluciferin probe with an APN inhibitor. Reprinted (adapted) with permission from Li et al. [41], copyright (2014) from American Chemical Society.

Bioluminescent enzyme substrates have also been studied with the goal of deep tissue imaging due to the enzyme-generated light. Two caged luciferin substrates, L-alanine-aminoluciferin and L-leucine-aminoluciferin, were designed to undergo a two-step process. First, the APN enzyme cleaved the substrate amide bond, and the liberated aminoluciferin was subsequently oxidized by firefly luciferase to produce light and enable detection of ovarian cancer in a tumor mouse model. The signal was also blocked when a small APN inhibitor was added (Figure 4) [41]. A very different approach used ^13^C NMR spectroscopy to monitor APN enzymatic activity. The APN catalyzed cleavage of an isotopically labeled substrate [1-^13^C]Ala-NH_2_ to produce [1-^13^C]Ala-OH which was monitored by a change in the ^13^C NMR chemical shift. This NMR method successfully measured APN enzyme activity in kidney homogenate and was selective for APN over other carboxypeptidases and dipeptidases [42]. It also displayed characteristics needed for hyperpolarized NMR experiments with a long spin-lattice relaxation time (*T*_1_) and a large *k*_cat_ value.

**Figure 5:**
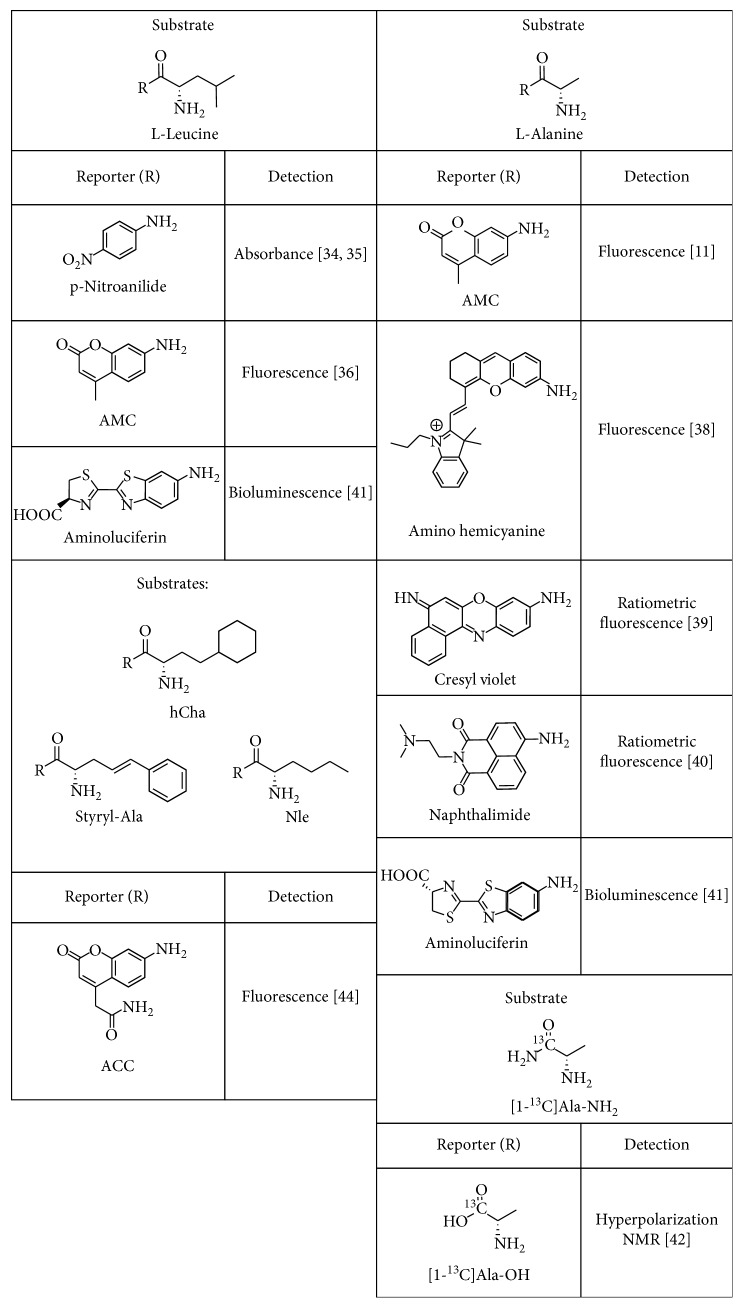
Reactive APN enzyme substrates with a reporter group and mode of detection.

APN is part of a large class of aminopeptidases, and the reactive substrates described above can also be recognized and cleaved by other aminopeptidases [30, 36, 43]. Thus, molecular imaging of APN would be improved by using a more specific reactive substrate or a set of substrates. A recent study of different aminopeptidases examined an array of natural and unnatural amino acid substrates and determined the specificity towards APN. The unnatural amino acids, styryl-Ala, hCha, and Nle (Figure 5), were found to have the highest selectivity for APN [44]. The results also suggested that an assay based on multiple substrates can produce a library fingerprint that identifies the responsible aminopeptidase more accurately than a single-substrate assay.

## 4. Nonreactive APN-Targeting Agents

### 4.1. Antibodies

Antibodies can also detect APN expression and are often used in immunohistochemistry to evaluate cell surface APN expression on cancerous tissue. In a clinical setting, this expression is a diagnostic biomarker to evaluate the stage of the disease and predict overall patient survival [2]. However, anti-APN antibodies exhibit a different specificity toward various glycosylated isoforms of APN. These glycosylation sites do not change the enzymatic activity of APN but do alter recognition by antibodies [45, 46]. A comparison was done with WM15, BF10, and 3D8 monoclonal antibodies where they detected APN expression in almost all tumor vasculature. However, the reactivity varied between the antibodies. The WM15 monoclonal antibody could also detect APN expression on intratumor and peritumor capillaries and thus, was determined to be the best APN detection tool [47]. However, this monoclonal antibody also targets vessels in the liver which limits its suitability for in vivo imaging or therapy. Interestingly, this antibody could detect APN expression on bone marrow-derived myeloid cells while a low molecular weight APN-targeting agent could not [48].

There is also a need to quantify APN expression on extracellular vesicles, but detection is difficult due to the small size of the vesicles and the low copy number. To address this issue, an in situ proximity ligation assay was developed. This technique utilizes a CD36 antibody coupled to a magnetic bead which binds to the extracellular vesicle. Then, APN and CD26 antibodies coupled to DNA oligonucleotides bind to their respective proteins on the vesicle, and a DNA circle is created to serve as a template for rolling circle amplification. Finally, oligonucleotides coupled to fluorophores are incorporated in the product to report the APN expression level [49].

### 4.2. Peptide-Based Targeting Agents

**Figure 6:**
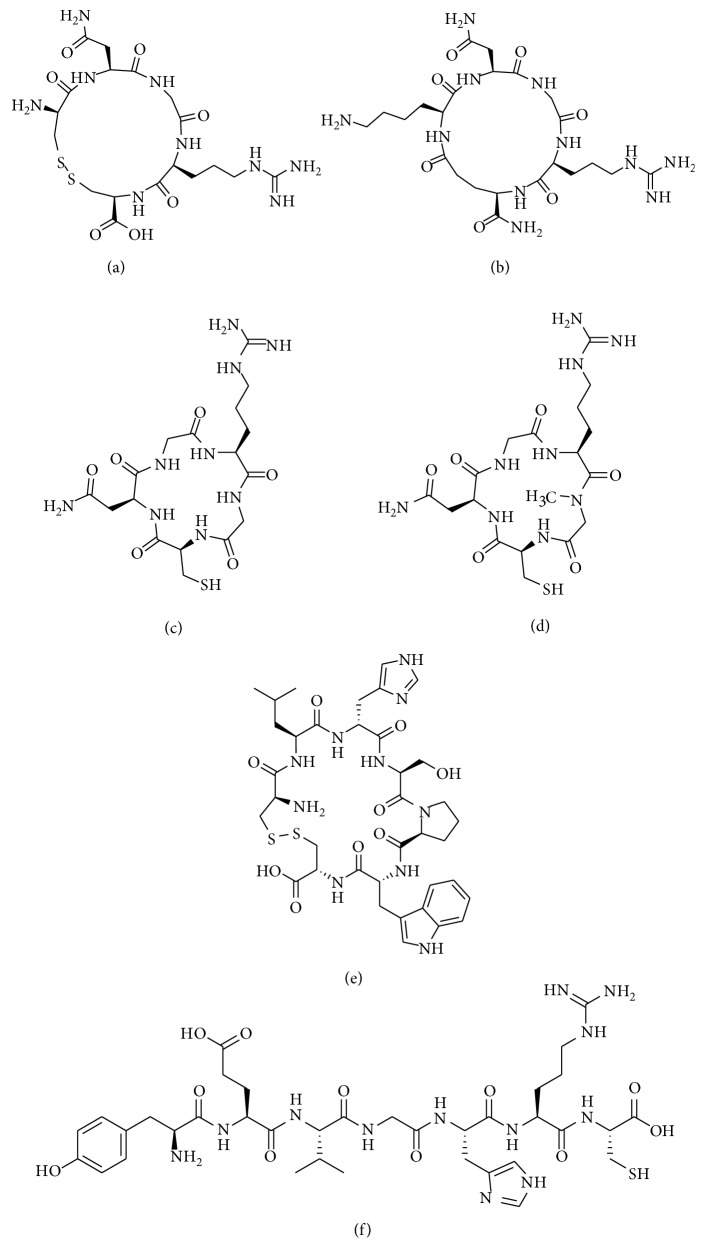
Nonreactive peptide-based APN-targeting agents: (a) cCNGRC; (b) cKNGRE; (c) coNGR; (d) cCGN_*N*Me_GRG; (e) cCLHSPWC; (f) peptide LN.

Studies have revealed peptide structures that can associate with the APN active site but are not cleaved by the enzyme. The best known peptide sequence, NGR (asparagine-glycine-arginine), was first discovered to target tumor vasculature in 1998 by injecting phage peptide libraries into nude mice bearing human breast carcinoma xenografts [50]. Since then, many molecular imaging probes have been created by utilizing the linear and cyclic version of the NGR peptide [51]. However, this review will only focus on the work done in the last five years by discussing the targeting agents shown in Figure 6.

**Figure 7:**
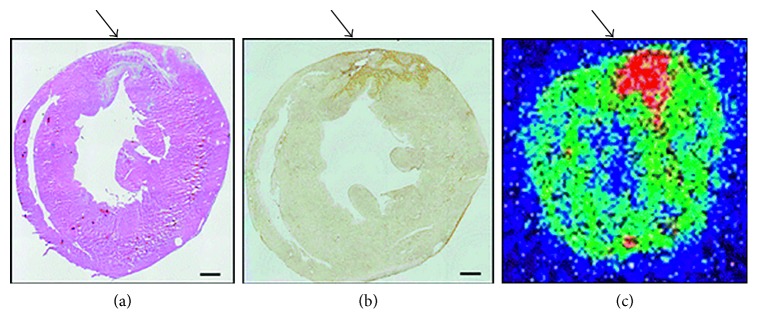
Increased in vivo uptake of ^68^Ga-cCNGRC into ischemic myocardium three days after myocardial ischemia and reperfusion. (a) H&E staining, (b) anti-APN antibody staining, and (c) ^68^Ga-cCNGRC PET imaging. Myocardial infarction is indicated by arrows, and scale bars are 1 mm. Reprinted with permission from Tillmanns et al. [60], copyright (2015).

The imaging performance of a linear NGR peptide has been directly compared to a cyclized version, cyclic CNGRC (cCNGRC), which incorporates a disulfide bond in the cyclic structure [52]. Since the cyclic version is more stable and has higher tumor targeting efficacy, it is used more often as a targeting unit on molecular probes. However, recent work using linear NGR as a targeting unit has shown successful accumulation in APN-positive lung carcinoma tumors, indicating that the linear sequence is effective in certain circumstances [53]. Studies have also used cCNGRC as a targeting agent for fluorescence and PET imaging in a pancreatic ductal adenocarcinoma xenograft model and well-differentiated heptacellular carcinoma, respectively [54, 55]. For PET imaging, three different ^68^Ga chelators conjugated to a cCNGRC targeting unit were compared. The uncharged NODAGA chelator outperformed DOTAGA and HBED and exhibited higher target to nontarget specificity [56]. A radiolabeled peptide drug conjugate was also created as a theranostic approach for imaging and targeted chemotherapy of melanoma. A chemotherapeutic drug, chlorambucil (CLB), was conjugated to cCNGRC and functionalized with a HYNIC chelator for radiolabeling with ^99m^Tc. The targeted ^99m^Tc-HYNIC-CLB-cCNGRC probe inhibited cancer cell growth, and its high hydrophilicity promoted rapid clearance from nontarget organs [57]. In an attempt to change the pharmokinectic profile, a PEG2 linker was added to the structure, but no change was observed [58]. A recent structural study showed that reporter groups attached to the Arg-Cys-COOH terminus of cCNGRC produced targeting agents with higher affinities for APN than analogues with reporter groups attached to the NH2-Cys-Asn terminus. These results are consistent with an X-ray crystal structure of a cCNGRC peptide bound to porcine APN and suggest that the carboxyl terminus of cCNGRC is the best site for conjugation [59]. With regard to angiogenesis in other diseases, cCNGRC probes have been used for cardiac healing. Using PET imaging, a radiolabeled cCNGRC probe targeted fibroblast and inflammatory cells in rats displaying myocardial ischemia and reperfusion (Figure 7) [60, 61]. Border and infarcted myocardium was visualized with a dual-isotope myocardial SPECT imaging method using ^111^In-DTPA-cCNGRC and ^99m^Tc-sestamibi. The overall uptake of ^111^In-DTPA-cCNGRC was higher in every section of the infarcted hearts compared to the healthy control hearts [62].

Importantly, there are two drawbacks for using cCNGRC as a molecular targeting agent. One is the susceptibility of the disulfide bond in the cyclic structure to undergo biodegradation or chemical modification. A specific concern is disulfide exchange with nearby thiols, a likely occurrence when many copies of the targeting unit are located on the surface of a nanoparticle. In order to create targeted liposomes that avoided the possibility of disulfide bridges between adjacent peptides, the cKNGRE targeting unit was developed as a cyclic peptide that lacked a disulfide linkage. Targeted liposomes coated with this peptide successfully delivered doxorubicin to cancer cells [63]. Furthermore, a radiolabeled version using the cKNGRE targeting unit enabled detection of renal tumors and metastases [64]. Another cyclized system that lacked a disulfide bond is coNGR which had higher stability than cCNGRC in blood and higher probe uptake in infarcted myocardium [65].

**Figure 8:**
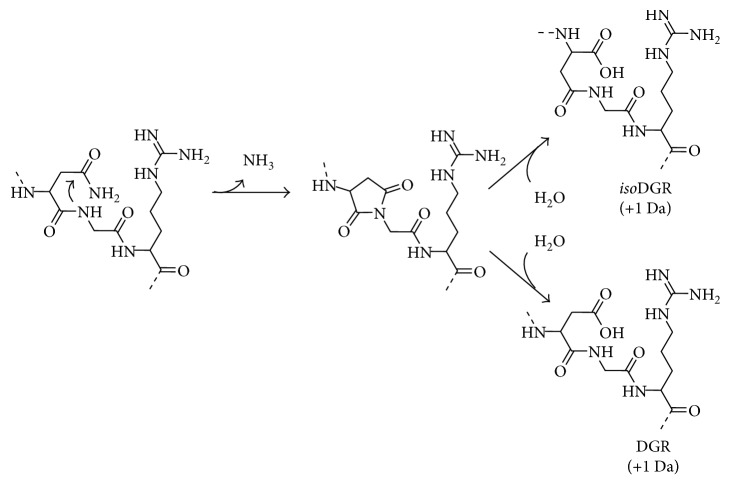
The peptide sequence NGR is susceptible to the spontaneous asparagine deamidation, which converts NGR into *iso*DGR and DGR. This occurs by a nucleophilic attack of the carbonyl group on the asparagine side chain by the nitrogen atom of the following glycine. This creates a succinimide intermediate which upon hydrolysis becomes *iso*DGR or DGR (with a molecular weight gain of 1 Da) [67, 68].

**Figure 9:**
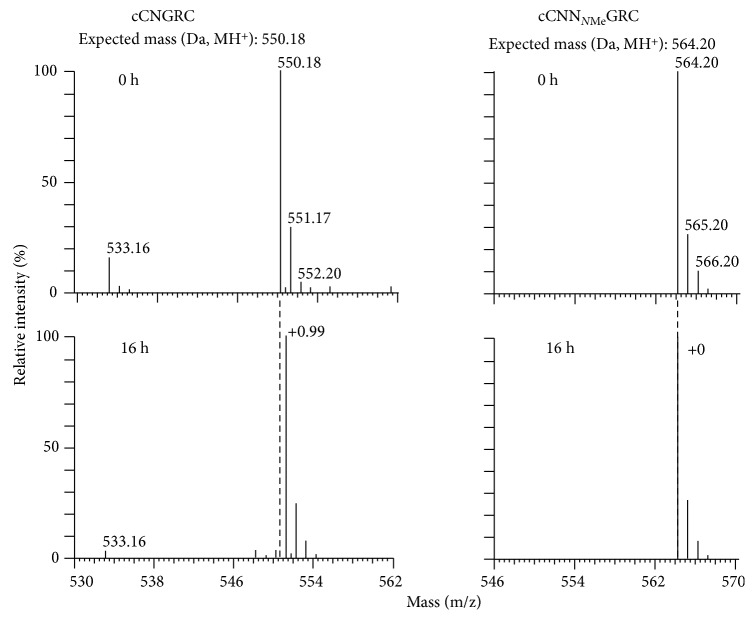
The cCN_*N*Me_GRC peptide is more stable than cCNGRC. For the cCN_*N*Me_GRC peptide, there was no change in molecular weight after a 16-hour incubation at 37°C in 0.1 M ammonium bicarbonate buffer, pH 8.5, whereas cCNGRC showed a molecular weight gain of 1 Da, indicating that the peptide underwent deamidation. Reprinted (adapted) with permission from Corti et al. [67], copyright (2017).

The second major drawback of the NGR sequence is its propensity to undergo spontaneous asparagine deamidation. In general, asparagine deamidation is a very slow process, but it is promoted when the asparagine is followed by a glycine residue and often further accelerated if the NGR sequence is within a cyclic structure [66]. As shown in Figure 8, the asparagine carbonyl within the NGR sequence is susceptible to nucleophilic attack by the adjacent glycine backbone nitrogen atom. This creates a succinimide intermediate that is hydrolyzed to form DGR or *iso*DGR, which are recognition motifs for various integrin receptors [69, 70]. Thus, over time, there is a switch in the targeting mechanism used by the probe to accumulate in tumors and vasculature undergoing angiogenesis [71]. This time-dependent change in structure and function of the NGR targeting unit can occur not only during storage but also throughout the course of an in vivo imaging experiment which is potentially problematic. Fortunately, a recent report indicates that this peptide stability problem can be solved by simply replacing the glycine residue in the NGR sequence with *N*-methylglycine [67]. Mass spectrometry studies showed that the modified peptide, cCN_*N*Me_GRC, was much more stable than cCNGRC. The spectra shown in Figure 9 indicate no change in molecular weight for cCN_*N*Me_GRC after 16 hours in 0.1 M ammonium bicarbonate buffer (pH 8.5), while the cCNGRC peptide gained 1 Da, indicating complete deamidation. Another stable methylated peptide, cCGN_*N*Me_GRG, targeted the pure APN enzyme with 15-fold higher potency than cCNGRC, and imaging studies with a radiolabeled version displayed higher tumor selectivity in melanoma-bearing mice over cCNGRC. Also, the cCGN_*N*Me_GRG peptide was an effective targeting agent when coated on the surface of TNF-bearing gold nanoparticles and liposomal doxorubicin.

In addition to NGR, other nonreactive peptide sequences have been found to target the APN enzyme. The linear peptide sequence YEVGHRC (peptide LN) was identified through a “one-bead-one-compound” approach using a microarray device. The linear peptide was used to create targeted liposomes for fluorescence imaging and therapy of a hepatocellular carcinoma tumor mouse model [72]. The cyclic peptide, cCLHSPWC, was also rationally designed to target and fluorescently label APN-positive prostate cancer cells. Furthermore, it displayed a higher therapeutic effect than cCNGRC in a prostate cancer mouse model [73].

### 4.3. Nonpeptide Targeting Agents

**Figure 10:**
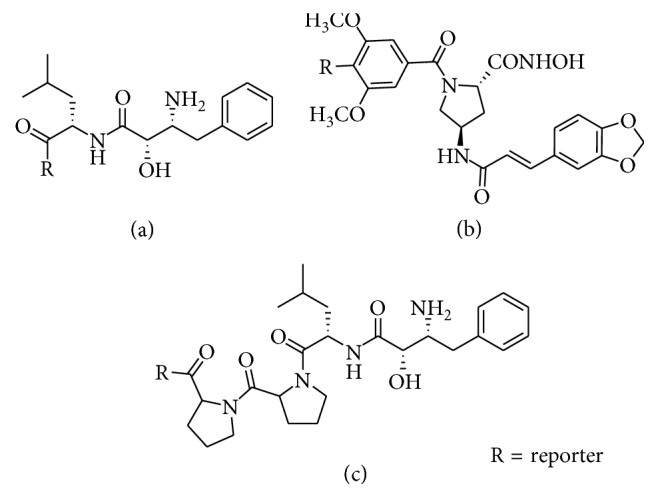
Nonpeptide APN-targeting agents: (a) bestatin; (b) I-23; (c) probestin.

Many nonpeptide molecules have been shown to bind to the APN active site and inhibit enzyme activity. These molecules include natural and synthetic products that induce cancer therapeutic effects such as inhibition of cancer cell migration, tumor angiogenesis, and tumor growth [74, 75]. Not surprisingly, some of these inhibitor molecules have been exploited as APN-targeting units for molecular imaging (Figure 10).

The best known small molecule APN inhibitor is bestatin. It was originally isolated from *Streptomyces oliboreticuli* MD976-C7 and was identified as a competitive, reversible inhibitor of APN enzymatic activity [76]. It is also approved in Japan as an adjuvant drug for treating patients with acute nonlymphocytic leukemia [77, 78]. Recently, a green fluorescent bestatin conjugate was prepared and used for fluorescence imaging of ovarian cancer [79]. A potential concern with this approach is low specificity for APN since bestatin is known to target twelve different aminopeptidases [80]. Probestin, a structurally related aminopeptidase inhibitor, was complexed to ^99m^Tc and shown to target APN-positive tumors, but with reversible binding [81]. An optimized structure produced a lower background in biodistribution studies, and the in vivo imaging performance was also evaluated [82, 83].

Over the years, many nonpeptide APN inhibitors have been synthesized, and the interested reader is directed to several excellent review articles [75, 84, 85]. Many of these APN inhibitors have zinc binding functional groups such as hydroxamate, carboxylate, sulfhydryl, sulfodiimide, or derivatives of phosphoric acid, that target the zinc cation in the APN active site [86]. In principle, they are all candidates for conversion into molecular imaging probes. However, currently, there has only been one reported case where a Cy5.5 fluorophore was conjugated to an APN inhibitor containing a hydroxamate group [84]. The fluorescent conjugate, called I-23, had similar in vitro APN affinity as bestatin with high potential for in vivo fluorescence imaging [87].

## 5. Multivalent Homotopic APN-Targeting Agents

**Figure 11:**
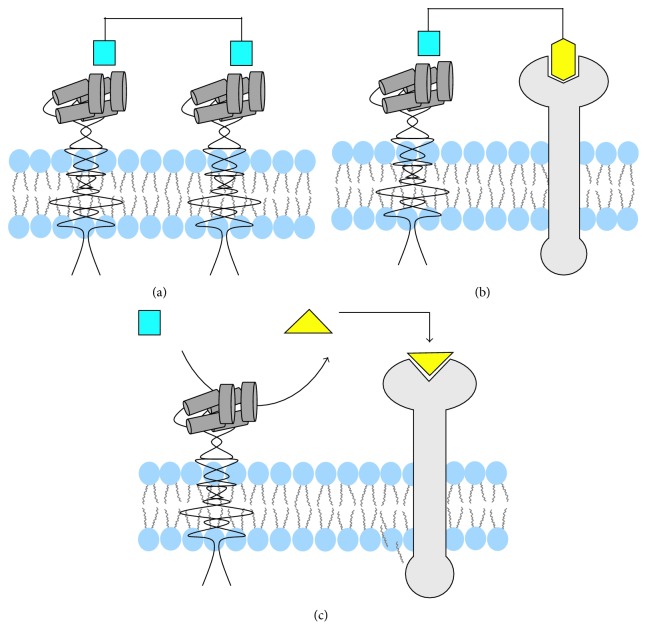
Multivalent targeting of the APN enzyme. (a) Bivalent homotopic targeting agents utilize the same targeting unit to cross-link two adjacent APN enzymes. (b) Bivalent heterotopic targeting agents bind to the APN enzyme and a nearby cell surface receptor. (c) Two-step activated targeting agents are cleaved by the APN enzyme, and then, the product binds a nearby cell surface receptor.

**Figure 12:**
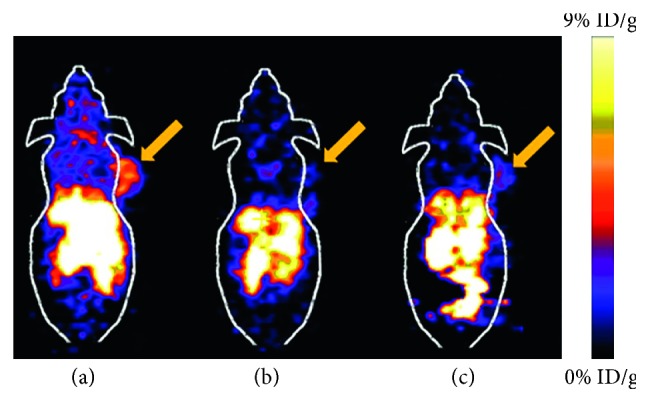
Comparison of a dimeric ^64^Cu-NGR probe uptake in an APN-positive tumor. (a) APN-positive tumor. (b) Blocked APN-positive tumor. (c) APN-negative tumor. Tumors are indicated by arrows. Reprinted (adapted) with permission from Chen et al. [89], copyright (2013) from American Chemical Society.

Multivalent homotopic molecular probes have multiple copies of the same targeting unit (Figure 11) [88]. To date, all efforts to create multivalent homotopic molecular probes for APN have employed the cNGR peptide as the targeting unit. In vitro APN binding studies have compared probes with one or two cNGR-targeting units (monovalent or divalent) where the divalent cNGR probe exhibited 2-fold higher APN avidity. The divalent probe also successfully targeted APN-positive tumors where targeting was blocked by excess unlabeled cNGR (Figure 12), and there was minimal uptake of the probe in APN-negative tumors [89, 90]. Furthermore, the divalent cNGR probe underwent increased cellular uptake with minimal cell efflux [89, 91]. In vivo studies showed increased tumor uptake of the divalent cNGR probe but with high probe accumulation in the liver and spleen compared to the monovalent probe. This is a known phenomenon where increasing the number of peptide ligands causes increased recognition by the reticuloendothelial system [92]. Multivalency has also been evaluated with other multivalent targeting agents such as cNGR nanoparticles and liposomes. As seen before, internalization of the probe correlated with the level of APN expression [93, 94]. Similar results were obtained when a study looked at in vivo fluorescence imaging of glioma [95, 96]. One study also compared targeted self-assembled nanoparticle systems with different surface loadings of cNGR. Interestingly, the nanoparticle system with the lower loading had higher tumor accumulation after 48 hours [97]. This result may be due to the close proximity of the targeting units, an effect that has been documented with related studies of cyclic RGD (arginine-glycine-aspartate) targeting to the *α*_v_*β*_3_ integrin receptor [98]; however, this hypothesis needs further testing. Additional studies have also compared tumor uptake of targeted cNGR-coated nanoparticles to untargeted nanoparticles. At 15 minutes to one hour after injection, active targeting exhibited higher tumor uptake than passive targeting, but after 24 hours, the opposite was seen [99]. The study highlights the large impact of passive targeting effects. Multivalency could also have an effect on receptor clustering as seen with other cell surface receptors such as the integrins [100]. When the APN enzyme was bound by anti-APN antibodies, the enzyme formed clusters and colocalized with caveolin 1. When cNGR-coated nanoparticles were used instead, the clustering event occurred at a faster rate, with higher colocalization and rapid internalization through caveolae-mediated endocytosis [101, 102].

Much less research has been conducted using multivalent APN probes for imaging angiogenesis. One study employed the coNGR-targeting unit described above which does not contain a disulfide linkage [65]. Monovalent and tetravalent coNGR probes were used to visualize infarcted myocardium with the dual administration of ^111^In-DTPA-cCNGRC and ^99m^Tc-sestamibi. The tetravalent coNGR probe produced less nonspecific targeting compared to the monovalent version. However, there was no significant difference between the two probes in targeting the myocardium infarcted regions. The authors suggested that the short spacer within the tetramer probe may be too short and rigid to enable bridging of multiple APN enzymes.

While a reasonable amount of work on multivalent cNGR probes has been published, the validity of some of the conclusions is clouded by the realization that the NGR motif is not stable and undergoes a spontaneous time-dependent deamidation to produce two different motifs with alternate targeting capabilities (Figure 8). Efforts have been made to attenuate the problem [71], but the recent discovery that deamidation can be eliminated by replacing the glycine residue in the sequence with *N*-methylglycine is very important [67]. It appears that systematic studies of multivalent NGR probes should be repeated with stable cCGN_*N*Me_GRG-targeting units in place of the less stable cCNGRC.

## 6. Multivalent Heterotopic Targeting Agents

Multivalent heterotopic molecular probes are equipped with different targeting units for different receptors (Figure 11). One study created the fusion protein, NGR-VEGI, with two recognition motifs: NGR that targets the APN enzyme and VEGI that inhibits vascular endothelial growth. The NGR-VEGI fusion protein exhibited significantly higher cell targeting and tumor uptake compared to the NGR peptide or VEGI protein alone [103]. It also exhibited cancer radiotherapeutic properties where ^188^Re-NGR-VEGI significantly reduced tumor volume with minimal off targeting [104]. A related study developed an MRI contrast agent where superparamagnetic nanoparticles were coated with RGD and NGR peptide units to target the *α*_v_*β*_3_ integrin receptor and APN, respectively. In comparison to nanoparticles coated with only one of the targeting peptides, the heterotopic agent accumulated in the tumor and increased the contrast-to-noise ratio [105]. This permitted MRI imaging of tumor angiogenesis using T2 weighting. Furthermore, a study used a fluorescent NGR/siRNA complex to target tumors that were APN negative and *α*_v_*β*_3_ integrin positive. The NGR peptide targeted the APN on tumor vasculature while the deamidated form, *iso*DGR, targeted *α*_v_*β*_3_ integrin receptors on tumor vasculature and tumor cells [106]. Another four-component targeting agent contained the cCNGRC peptide, the RGD peptide, an aggregation-induced emission luminogen, and a nuclear localization signal. Competitive cell uptake experiments showed that the probe was selective for cells that overexpressed both the *α*_v_*β*_3_ integrin receptor and APN over cells that expressed only one of the proteins. After cell uptake, the probe fluorescence was turned on due to localization and aggregation in the nucleus. The targeting agent remained in the cells over several passages, which enabled long-term tracing in living cells [107].

Finally, it is worth noting a newly developed two-step target activation approach. As shown in Figure 11, a caged probe is cleaved by the APN enzyme, to reveal a targeting motif that subsequently associates with a neighboring receptor. The concept was demonstrated with the iNGR system which contains the cyclized peptide sequence CRNGRGPDC. The APN enzyme recognizes the NGR sequence and holds the targeting agent in place while a hydrolytic enzyme on the cell surface cleaves off the GPDC sequence. The remaining peptide sequence, CRNGR, binds to neuropilin-1 and is internalized. This targeting system has been successful in targeting and imaging glioblastoma and colorectal tumors [108, 109].

## 7. Conclusion and Future Directions

The two most common protein targets for molecular imaging of cancer and angiogenesis are integrin receptors and aminopeptidases. Integrin receptors are heterodimer membrane glycoproteins that are essential for fetal development, wound healing, and growth and development [110]. They aid in cancer invasion and migration by degrading the extracellular matrix and have been used for imaging of myocardial infarction [111], rodent models of the hind limb ischemia [112], and atherosclerotic plaques [113]. In comparison, aminopeptidases are enzymes that cleave reactive substrates and thus, can release an amplified number of imaging reporter groups. This makes them very attractive targets for molecular imaging. Upregulation of the APN enzyme is associated with multiple cancers along with myocardial ischemia and angiogenesis. However, targeting this enzyme for imaging has been challenging due to its overlap in substrate recognition with other aminopeptidases [30, 36, 43]. This problem is currently being addressed by efforts to optimize the substrate structure for high APN specificity. In the last five years, advances in the development of APN reactive substrates have included in vivo optical imaging using NIR fluorescent probes for deeper tissue penetration, bioluminescence probes that illuminate their own light, and ratiometric probes that attenuate environmental effects. One promising clinical application is to use these probes for fluorescence-guided surgery where the surgeon aims at resecting cancerous tissue with negative margins and thus improving patient outcome [37, 114, 115].

APN nonreactive targeting agents can be divided into three categories: antibodies, peptides, and nonpeptides. Antibodies are very good at distinguishing APN isoforms with different glycosylation sites. Peptide targeting agents have primarily focused on the NGR motif, but there is a problem with this sequence due to structural instability from a spontaneous deamidation reaction [113]. A recent breakthrough may have solved the problem by substituting glycine with *N*-methylglycine [67]. There are many nonpeptide inhibitors of APN, although, only a small number have been converted into probes for imaging [87]. Furthermore, molecular imaging of APN has mainly focused on targeting cancerous tissue, tumor angiogenesis, and angiogenesis in myocardial infarction. Future studies could potentially investigate other cardiovascular diseases related to angiogenesis such as atherosclerosis and peripheral artery disease. Presently, it is unknown if the APN isoform associated with these other cardiovascular tissues can be recognized by the same molecular probes discussed above. Tissue-specific signaling processes may have to be activated to switch APN to the open conformation that allows for substrate binding [7].

Overall, selective molecular targeting of APN enzyme is challenging due to the overlap in substrate recognition with other aminopeptidases. But over the last five years, new classes of reactive APN substrates and nonreactive targeting agents have been developed for improved imaging of cancer and angiogenesis. It seems likely that APN imaging can be expanded to detect and monitor other diseases that are associated with angiogenesis.
